# Tacrolimus Treatment for TAFRO Syndrome

**DOI:** 10.3390/biomedicines12051070

**Published:** 2024-05-12

**Authors:** Taiichiro Shirai, Shinya Ichikawa, Jun Saegusa

**Affiliations:** 1 Department of Rheumatology and Clinical Immunology, Kobe University Graduate School of Medicine, Kobe 650-0017, Japan; 2 Laboratory of Immune Response Dynamics, WPI Immunology Frontier Research Center, Osaka University, Osaka 565-0871, Japan; 3 Department of Immune Response Dynamics, Research Institute for Microbial Diseases, Osaka University, Osaka 565-0871, Japan

**Keywords:** TAFRO syndrome, idiopathic multicentric Castleman disease (iMCD), iMCD-TAFRO, tacrolimus, calcineurin inhibitor

## Abstract

TAFRO syndrome is an acute systemic inflammatory disorder characterized by thrombocytopenia, anasarca, fever, reticulin myelofibrosis, renal dysfunction, and organomegaly. While its lymph node pathology is similar to that of idiopathic multicentric Castleman disease (iMCD), the clinical features of TAFRO syndrome differ from those of typical iMCD, as they include a more aggressive clinical course and high mortality. However, an optimal treatment strategy for TAFRO syndrome has not yet been established, owing to a poor understanding of its pathogenesis. The limited cases we encountered suggest that tacrolimus treatment in combination with glucocorticoids may potentially be effective and well tolerated as an initial treatment, and hold promise as a glucocorticoid-sparing agent. Herein, we report an additional case and review the sparse literature available regarding TAFRO syndrome treated via tacrolimus.

## 1. Introduction

Castleman disease (CD) is a rare lymphoproliferative disorder that involves single (unicentric) or multiple (multicentric) lymph nodes [1]. Multicentric CD (MCD) is further subdivided into human herpesvirus type-8 (HHV-8)-associated MCD; polyneuropathy, organomegaly, endocrinopathy, monoclonal plasma cell disorder, and skin changes (POEMS) syndrome-associated MCD; and idiopathic MCD (iMCD) [2]. In 2010, Takai et al. first reported three Japanese cases with fever, thrombocytopenia, pleural effusion and ascites, hepatosplenomegaly, and reticulin fibrosis of the bone marrow, proposing a new disease entity [3]. The systemic inflammatory disorder with a constellation of symptoms such as thrombocytopenia, anasarca (edema, pleural effusion, and ascites), fever, reticulin myelofibrosis, renal dysfunction, and organomegaly (hepatosplenomegaly and lymphadenopathy) was named TAFRO syndrome, derived from the acronym of its symptoms [4]. An epidemiologic analysis of TAFRO syndrome in Japan estimated an annual incidence rate of 0.9–4.9 per million [5]. The etiology of TAFRO syndrome is undetermined; however, patients with this syndrome often have generalized lymphadenopathy, and their lymph node pathologies are typically similar to that of iMCD, classified into hypervascular, plasma cell (or plasmacytic), and mixed types [2,6]. Therefore, TAFRO syndrome is sometimes considered a subtype of iMCD (iMCD-TAFRO), whereas iMCD without TAFRO syndrome is described as “not otherwise specified” (iMCD-NOS) [2]. However, the nature of iMCD-TAFRO is different from that of iMCD-NOS, with a more aggressive clinical course and high mortality rate that is associated with refractory ascites and thrombocytopenia [7,8]. These unique clinical and laboratory features suggest that iMCD-TAFRO is distinct from iMCD.

Accumulating evidence on the pathogenesis of iMCD suggests that aberrant interleukin-6 (IL-6) signaling is a key driver of iMCD-NOS [9]. IL-6 is a multifunctional pro-inflammatory cytokine that induces B-cell and plasma cell maturation, leading to increased immunoglobulin production, and stimulates megakaryocyte maturation, typically resulting in thrombocytosis [10]. The intensity of iMCD-NOS symptoms correlates with serum IL-6 levels, which can be highly elevated during flare-ups [11]. However, in iMCD-TAFRO, IL-6 is only mildly elevated, and the typical clinical features of iMCD-NOS associated with excess IL-6, such as polyclonal hypergammaglobulinemia and thrombocytosis, are absent [8]. This suggests that IL-6 may not be the primary pathological driver of iMCD-TAFRO and that its exact pathophysiology remains to be elucidated. Currently, the international consensus treatment guidelines recommend anti-IL-6 therapy (tocilizumab or siltuximab) as the first-line treatment for iMCD-TAFRO [12]. However, evidence of an association between serum IL-6 levels and the efficacy of anti-IL-6 therapies for treating iMCD-TAFRO remains limited [8]. Treatment failure with anti-IL-6 therapy has been reported in 50% of iMCD-TAFRO cases [12]. Therefore, optimal treatment strategies for TAFRO syndrome are urgently needed.

Cyclosporine A, a calcineurin inhibitor, has been used to treat TAFRO syndrome, particularly for improving persistent ascites and thrombocytopenia [13,14,15,16,17]. Notably, this treatment may be useful in cases of TAFRO syndrome that are refractory to anti-IL-6 therapy [14,15]. Cyclosporine A exerts an immunosuppressive effect by inhibiting the dephosphorylation of the nuclear factors of activated T-cells, by binding to calcineurin [18,19]. Tacrolimus is another calcineurin inhibitor, and we previously reported the first cases of TAFRO syndrome successfully treated with tacrolimus, including one case that was refractory to anti-IL-6 therapy and intolerant to cyclosporine A [20]. Herein, we report an additional case of TAFRO syndrome effectively treated with tacrolimus and glucocorticoids, in which tacrolimus was also useful as a glucocorticoid-sparing agent. Moreover, we review the literature on TAFRO syndrome treated with tacrolimus and explore the efficacy of this approach.

## 2. Case Presentation

**Figure 1:**
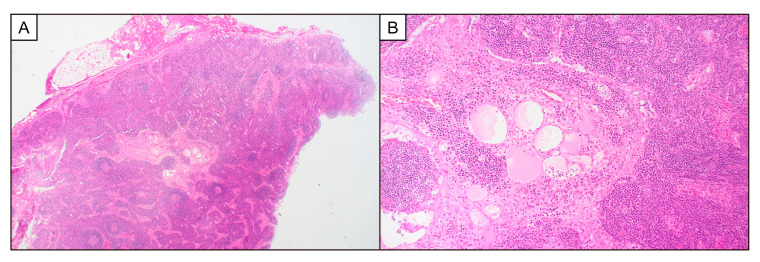
Histopathology of the cervical lymph node. The expansion of the interfollicular zone with vascular proliferation and increased plasma cells is observed. Representative images of hematoxylin and eosin-stained sections at 20× (**A**) and 100× (**B**) magnification are shown.

A 33-year-old Japanese woman with no prior medical history was referred to a hospital with a 3-week history of abdominal distension and a fever of 38.2 °C. Upon physical examination, the patient exhibited swollen cervical and axillary lymph nodes (diameter: 1 cm), mild abdominal tenderness, and pitting edema of the lower legs. Laboratory studies revealed thrombocytopenia (platelet count, 65,000/μL [reference range, 158,000–358,000/μL]); anemia (hemoglobin concentration, 7.8 g/dL [reference range, 11.6–14.8 g/dL]); the absence of hypergammaglobulinemia (serum immunoglobulin G concentration, 1137 mg/dL [reference range, 861–1747 mg/dL]); elevated serum levels of alkaline phosphatase (919 U/L [reference range, 106–322 U/L]), C-reactive protein (CRP, 17.22 mg/dL [reference range, 0.00–0.14 mg/dL]), soluble IL-2 receptor (sIL-2R, 1702 U/mL [reference range, 122–496 U/mL]), and IL-6 (55.0 pg/mL [reference range, 0.0–7.0 pg/mL]); elevated plasma levels of vascular endothelial growth factor (VEGF, 297.0 pg/mL [reference range, 0.0–38.3 pg/mL]); and mild renal dysfunction (estimated glomerular filtration rate, eGFR; 58 mL/min/1.73 m^2^) with microhematuria. The test results for autoantibodies such as anti-double-stranded DNA and anti-neutrophil cytoplasmic antibodies, as well as viruses such as HHV-8, human immunodeficiency virus, and Epstein–Barr virus, were negative. Computed tomography (CT) revealed mild cervical and axillary lymphadenopathy (1–1.5 cm in diameter), bilateral pleural effusions, ascites, and hepatosplenomegaly. Cervical lymph node biopsy revealed the expansion of the interfollicular zone with vascular proliferation and plasmacytic infiltrates (Figure 1). A bone marrow biopsy revealed hyperplasia of the megakaryocytes and reticulin fibrosis. We found no evidence of infectious, malignant, or autoimmune diseases that can mimic TAFRO syndrome [8,21,22]. Additionally, the diagnosis of hemophagocytic lymphohistiocytosis (HLH) was not established according to the HLH-2004 criteria [23] or the HScore for reactive hemophagocytic syndrome [24]. Thus, the patient met the diagnostic criteria for TAFRO syndrome proposed by both the Japanese TAFRO Syndrome Research Team [21,25] and the Castleman Disease Collaborative Network [8]. The disease severity was estimated to be slightly severe (grade 3 out of 5) according to the 2019 updated disease severity classification for TAFRO syndrome [21].

**Figure 2:**
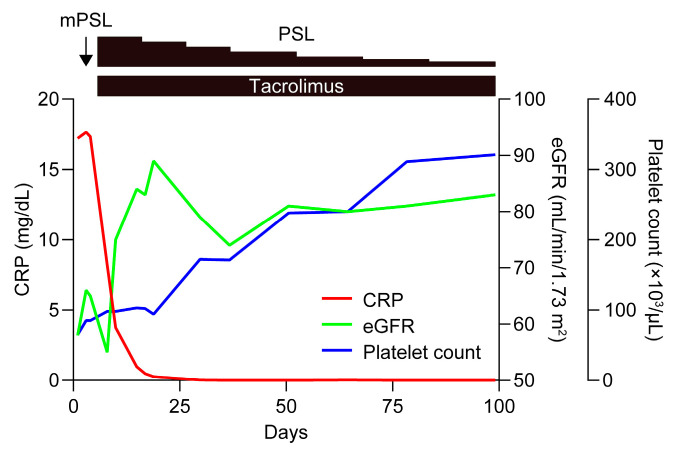
Clinical course of the patient. After the diagnosis of TAFRO syndrome, mPSL pulse therapy was initiated at 1000 mg/day for three consecutive days, followed by 60 mg/day (1 mg/kg/day) of oral PSL and 4 mg/day (0.07 mg/kg/day) of tacrolimus, targeting a trough concentration of 5–10 ng/mL. This reduced the serum CRP levels and improved thrombocytopenia and renal dysfunction. Tacrolimus treatment was continued, and the PSL was tapered.

Half of the cases of TAFRO syndrome are reported to be unresponsive to anti-IL-6 therapy [12], and we have previously reported cases of TAFRO syndrome successfully treated with tacrolimus that presented thrombocytopenia and ascites with less severe disease activity, one of which proved refractory to anti-IL-6 therapy and resulted in septic shock [20]. In light of these facts, after obtaining informed consent from the patient, we initiated methylprednisolone (mPSL) pulse therapy at 1000 mg/day for three consecutive days, followed by 60 mg/day (1 mg/kg/day) of oral PSL and 4 mg/day (0.07 mg/kg/day) of tacrolimus, targeting a trough concentration of 5–10 ng/mL (Figure 2). This alleviated the patient’s fever, thrombocytopenia, and renal dysfunction. The abdominal distension also gradually subsided, and CT showed the complete resolution of the bilateral pleural effusions and ascites after 4 weeks of treatment. Tacrolimus was continued, and glucocorticoids were tapered and eventually discontinued. The patient has had an uneventful course without relapse for >4 years.

## 3. Discussion

Although a standard treatment strategy for TAFRO syndrome has not yet been established, we report an additional case of TAFRO syndrome successfully treated with tacrolimus. Our findings suggest that tacrolimus in combination with glucocorticoids may be an effective and well-tolerated initial treatment for TAFRO syndrome, while showing promise as a glucocorticoid-sparing agent. According to a recent surveillance study in Japan, most patients with TAFRO syndrome receive glucocorticoids as a first-line treatment [13]. Tocilizumab, cyclosporine A, and rituximab (an anti-CD20 antibody for B-cell depletion) are commonly used to treat glucocorticoid-resistant patients [13]. However, despite these immunosuppressive treatments, the mortality rate remains high for patients with this syndrome [7]. Furthermore, in some cases, severe infections occur during the treatment, which can be fatal according to our review of the literature (Table 1). These facts highlight a need for the development of specific therapeutic strategies.

A challenge in finding optimal treatments for TAFRO syndrome is the incomplete understanding of its pathogenesis. Previous studies have shown distinct proteomic profiles for iMCD-TAFRO and iMCD-NOS, suggesting that diverse driving factors underlie the iMCD disease spectrum [51]. In this context, emerging evidence points to activated T-cells as potential pathogenic drivers of iMCD-TAFRO. Iwaki et al. reported that C-X-C motif chemokine ligand 10 (CXCL10), also known as interferon γ-induced protein 10, exhibited higher levels in patients with iMCD-TAFRO compared to those with iMCD-NOS during flare-ups [52]. CXCL10 plays an important role in recruiting activated T-cells to inflammation sites [53]. Fajgenbaum et al. found that sIL-2Rα, a marker of T-cell activation, and VEGF-A were upregulated during flare-ups in patients with iMCD-TAFRO who were refractory to anti-IL-6 therapy. Additionally, these patients exhibited increased numbers of activated CD8^+^ T-cells [54]. Serum proteomic analysis revealed the enrichment of the phosphatidylinositol-3 kinase/Akt/mammalian target of rapamycin (mTOR) pathway in these cases, and sirolimus—an mTOR inhibitor—proved to be effective [54]. Furthermore, the symptoms of capillary leak syndrome, an adverse effect of IL-2 immunotherapy, reveal some overlap with those of TAFRO syndrome, suggesting the possible contribution of IL-2 to its pathogenesis [14].

Given the proposed pathogenesis, treating TAFRO syndrome with calcineurin inhibitors such as cyclosporine A and tacrolimus, which target activated T-cells and inhibit the secretion of proinflammatory cytokines (particularly IL-2), emerges as a rational therapeutic strategy. Although cyclosporine A has been widely used in the treatment of TAFRO syndrome [13], information regarding cases treated using tacrolimus remains limited. To the best of our knowledge, only four studies have reported the use of tacrolimus treatment for TAFRO syndrome [20,38,55,56] (Table 2), excluding one in which the duration of tacrolimus use was markedly short to evaluate its efficacy [33]. Among these studies, we first described two cases of TAFRO syndrome that were successfully treated using tacrolimus [20].

The first case [20] is a 68-year-old Japanese woman with a 4-week history of abdominal distension and a fever of 38.1 °C. Laboratory studies revealed anemia, thrombocytopenia (platelet count, 38,000/μL), elevated serum levels of CRP (2.70 mg/dL), and renal dysfunction (eGFR, 25.5 mL/min/1.73 m^2^). CT showed generalized lymphadenopathy (1–1.5 cm in diameter), bilateral pleural effusion, massive ascites, and hepatosplenomegaly. The histopathological findings for the lymph nodes were compatible with the mixed type of CD. Moreover, reticulin fibrosis and megakaryocytic hyperplasia were found in the bone marrow. These findings met the diagnostic criteria for TAFRO syndrome, and the disease severity was estimated to be slightly severe (grade 3 out of 5). Oral PSL at 60 mg/day (1 mg/kg/day) improved the renal dysfunction but did not resolve the thrombocytopenia and massive ascites. Additional treatment with tocilizumab failed to improve the symptoms, but rather the patient developed septic shock due to empyema, necessitating the discontinuation of tocilizumab. Moreover, cyclosporine A was discontinued within a week due to its hepatotoxicity. Therefore, we initiated tacrolimus at 4 mg/day (0.07 mg/kg/day), targeting a trough concentration of 5–10 ng/mL, which dramatically alleviated thrombocytopenia and ascites. With the use of tacrolimus, glucocorticoids were successfully tapered. The patient has had an uneventful course without relapse for >6 years.

**Figure 3:**
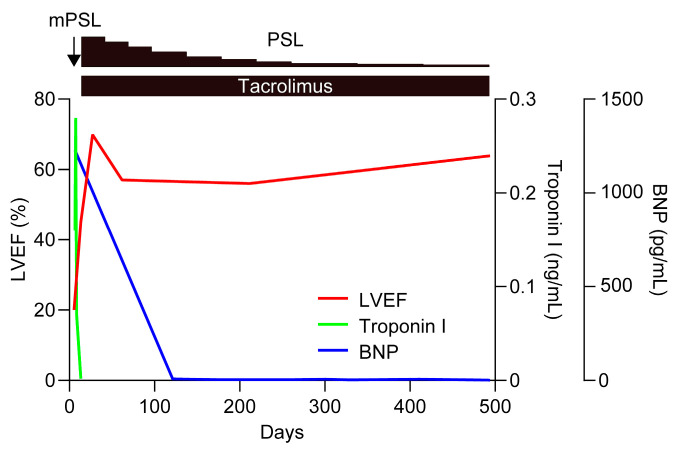
Clinical course of cardiomyopathy in TAFRO syndrome. After the sudden onset of cardiogenic shock, mPSL pulse therapy was initiated at 1000 mg/day for three consecutive days, followed by 60 mg/day (1 mg/kg/day) of oral PSL and 4 mg/day (0.07 mg/kg/day) of tacrolimus, targeting a trough concentration of 5–10 ng/mL. Serial echocardiography showed an improvement in LVEF. The levels of plasma BNP and serum troponin I were consistently reduced. Tacrolimus treatment was continued, and the PSL was tapered. BNP, brain natriuretic peptide; LVEF, left ventricular ejection fraction.

The second case [20] is a 17-year-old Japanese man with a 4-week history of abdominal pain and a fever of 38.8 °C. His progressive symptoms included anemia, thrombocytopenia (platelet count, 68,000/μL), elevated serum levels of CRP (23.00 mg/dL), and renal dysfunction (eGFR, 50.6 mL/min/1.73 m^2^) with microhematuria. CT revealed mild cervical and axillary lymphadenopathy (1–1.5 cm in diameter), bilateral pleural effusion, ascites, and hepatosplenomegaly. The histopathological findings of the lymph nodes were compatible with the mixed type of CD, and the reticulin fibrosis and normoplasia of megakaryocytes were found in the bone marrow. A diagnosis of TAFRO syndrome was made and the disease severity was estimated to be slightly severe (grade 3 out of 5). During the course of the disease, the patient presented with the sudden onset of cardiogenic shock without evidence of takotsubo cardiomyopathy, myocardial infarction, or viral myocarditis. Echocardiography showed the severe and diffuse hypokinesis of both ventricles and moderate pericardial effusion. In addition to mPSL pulse therapy at 1000 mg/day for three consecutive days and 60 mg/day (1 mg/kg/day) of oral PSL, we started 4 mg/day (0.07 mg/kg/day) of tacrolimus, targeting a trough concentration of 5–10 ng/mL. This improved the symptoms of TAFRO syndrome, including thrombocytopenia and anasarca, and repeat echocardiography revealed normal wall motion without pericardial effusion 4 weeks after initiation of the treatment (Figure 3). Tacrolimus was well tolerated and continued while the glucocorticoids were tapered and eventually discontinued. The patient has had an uneventful course without relapse for >5 years.

In all three cases we encountered, including the present one, tacrolimus in combination with glucocorticoids was effective as induction therapy for the symptoms of TAFRO syndrome, including, but not limited to, thrombocytopenia and ascites. Additionally, tacrolimus was useful as a glucocorticoid-sparing agent. Considering the uneventful course without relapse for several years observed in these patients, tacrolimus treatment alone or with low-dose glucocorticoids could potentially serve as maintenance therapy for TAFRO syndrome. The disease severity of TAFRO syndrome in these patients was estimated to be slightly severe (grade 3 out of 5) according to the 2019 updated disease severity classification [21]. While the precise type of patients or specific disease symptoms that were most effectively targeted by tacrolimus remain unclear, treatment with tacrolimus might be particularly effective in cases with thrombocytopenia and ascites, especially when the disease activity is not severe.

As described above, we reported cardiomyopathy, a rare complication of TAFRO syndrome, which was reversed upon treatment with tacrolimus and glucocorticoids. To date, cardiomyopathy in TAFRO syndrome has been reported in only a few cases [57,58,59], one of which was refractory to anti-IL-6 therapy, suggesting that other proinflammatory cytokines may be involved [57]. Given the documented association between high-dose IL-2 immunotherapy and reversible cardiomyopathy [60], and our case’s successful treatment outcome with tacrolimus, IL-2 might contribute to cardiomyopathy in TAFRO syndrome, although the mechanism remains poorly understood.

In addition to adult cases, a preprint study by Goteti et al. described a case of TAFRO syndrome in an infant treated with tacrolimus [55]. The patient was initially treated with tocilizumab and glucocorticoids. However, the anasarca persisted; therefore, tacrolimus was administered, which proved to be effective. The tacrolimus was continued without adverse events and the glucocorticoids were successfully tapered [55]. This case suggests that tacrolimus may also be effective and well tolerated in infants with TAFRO syndrome.

Conversely, two other reports have described cases of TAFRO syndrome where tacrolimus was ineffective. Nagai et al. reported a possible recurrent case of TAFRO syndrome that developed in a kidney transplant recipient who was taking daily immunosuppressants, including tacrolimus [56]. The patient was treated with tacrolimus and mycophenolate mofetil, in addition to glucocorticoids; however, this proved ineffective, and the patient eventually died [56]. Considering the possibility of multiple pathogeneses of TAFRO syndrome, including T-cell and B-cell/plasma cell-dominant variants, it may be reasonable to consider rituximab or tocilizumab as first-line treatments instead of tacrolimus, particularly in patients who are already taking tacrolimus at the onset of TAFRO syndrome. Moreover, Abe et al. reported cases of TAFRO syndrome that were refractory to tacrolimus treatment but were effectively treated with cyclosporine A [38]. Both cyclosporine A and tacrolimus exert their immunosuppressive effects by binding to calcineurin on activated T-cells; however, they bind to different binding proteins, cyclophilins, and FK506-binding proteins, respectively [18,19]. This may explain the differences in their efficacy levels and adverse events in individual cases of TAFRO syndrome. In this regard, cyclosporine A exhibits hepatotoxicity during the treatment of some cases (Table 1), and thus replacing cyclosporine A with tacrolimus may be beneficial.

## 4. Conclusions

Given that TAFRO syndrome is a relatively newly recognized disease of unknown etiology, information on the best therapeutic strategies is limited. Currently, some molecular targeted therapies, including tocilizumab, siltuximab, and rituximab, have been used; however, their efficacy is not satisfactory. Herein, we report an additional case and review the existing literature on tacrolimus treatment for TAFRO syndrome. Although based on a small number of cases, treatment with tacrolimus in combination with glucocorticoids may be potentially effective and well tolerated in TAFRO syndrome, especially in cases presenting with thrombocytopenia and ascites with less severe disease activity. In addition, tacrolimus treatment may be useful as a glucocorticoid-sparing agent and as a maintenance therapy in TAFRO syndrome. One of the major hurdles in developing optimal treatment strategies for TAFRO syndrome is the difficulty associated with understanding its pathogenesis. Again, the efficacy of tacrolimus treatment discussed here is based on a limited number of cases, with reports on tacrolimus-refractory cases. Considering the possibility that multiple factors drive TAFRO syndrome, further research is needed to determine the specific patients and symptom profiles that will respond to tacrolimus. Ultimately, gaining a comprehensive understanding of the pathogenesis of TAFRO syndrome would provide insight into the rationale of tacrolimus treatment and contribute to the development of optimal therapeutic strategies.

## Figures and Tables

**Table 1:** Adverse events associated with treatments for TAFRO syndrome.

Treatments (Suspected Drugs)	Adverse Events			
Monotherapy				
Glucocorticoid	Bacterial infection [26,27,28,29]	CMV infection [3,28,29,30,31]	Fungal infection [29]	Tuberculosis [32]
Tocilizumab	Bacterial infection [29]	Toxic epidermal necrolysis [33]		
Cyclosporine A	Hepatotoxicity [20,30,34,35,36]	Renal toxicity [37]	Thrombotic microangiopathy [38]	
Combination therapy with glucocorticoid				
Tocilizumab	Bacterial infection [20,39,40,41,42,43]	CMV infection [37,43,44,45,46]	Fungal infection [40,42]	
Cyclosporine A	Bacterial infection [47]	CMV infection [32,47,48]	Fungal infection [14,17,47]	
Rituximab	Bacterial infection [49,50]	CMV infection [50]		

CMV, cytomegalovirus.

**Table 2:** Clinical characteristics and outcomes of patients with TAFRO syndrome treated with tacrolimus.

Patient	1 (Present Case)	2 [20]	3 [20]	4 [55]	5 [56]	6 [38]	7 [38]
Clinical characteristics							
Disease new-onset or relapse	New onset	New onset	New onset	New onset	Relapse	New onset	Relapse
Sex	Female	Female	Male	Female	Male	Female	Male
Age at disease onset or relapse	33	68	17	11 months	57	47	64
Disease severity classification [21]	3	3	3	2	2	4	2
Treatment history							
Previous treatment	N.A.	N.A.	N.A.	N.A.	PSL, TAC, MMF	N.A.	PSL
Treatment for initial onset or relapse							
mPSL/PSL	+	+	+	+	+	+	+
Experienced agents	TAC	TCZ, CsA, TAC	TAC	TCZ, TAC	TAC, MMF	TCZ, TAC, MMF, CsA	TAC, CsA
Effective agents	TAC	TAC	TAC	TCZ, TAC	−	CsA	CsA
Adverse events	−	TCZ: bacterial infection CsA: hepatotoxicity	−	−	−	−	−
Relapse-free survival	>4 years	>6 years	>5 years	>1.5 years	−	N.A.	N.A.

TAC, tacrolimus; TCZ, tocilizumab; CsA, cyclosporine A; MMF, mycophenolate mofetil; N.A., not available.

## Data Availability

The raw data supporting the conclusions of this article will be made available by the authors upon request.
